# Cracking the Left Main Coronary Artery Nodular Calcium With a Scoring Balloon

**DOI:** 10.7759/cureus.44123

**Published:** 2023-08-25

**Authors:** Dibyasundar Mahanta, Debasis Acharya, Debasish Das

**Affiliations:** 1 Cardiology, Sunshine Hospital, Bhubaneswar, IND; 2 Cardiology, All India Institute of Medical Sciences (AIIMS), Bhubaneswar, IND

**Keywords:** myocardial infarction, left main coronary artery, scoring balloon, nodular calcium, cracking

## Abstract

Nodular calcium in the coronary artery poses a great challenge during coronary intervention. Although rotational atherectomy is the preferred modality of treatment of nodular calcium, we treated the left main coronary artery (LMCA) nodular calcium with a scoring balloon during primary angioplasty of an octogenarian with anterior wall myocardial infarction and EKG suggestive of LMCA occlusion. During primary coronary angioplasty, the scoring balloon alone also sometimes suffices in achieving good angiographic results and thrombolysis in myocardial infarction grade III (TIMI III) flow.

## Introduction

Coronary artery calcium and thrombus pose significant challenges during the coronary intervention, especially during the left main coronary intervention. Dealing with nodular calcium in the elderly presents challenges during interventions, as these are resistant to cracking [1]. Aggressive high-pressure dilatation (>35 atm pressure) can lead to vessel dissection and perforation, and in some cases, it results in inadequate stent expansion with suboptimal angiographic outcomes. Dealing with such tenacious calcium deposits during left main coronary intervention poses a great challenge, as patients are frequently hemodynamically unstable, experiencing secondary ischemic ventricular arrhythmia, and requiring multiple inotropic supports.

We present a case of a successful left main coronary artery (LMCA) angioplasty involving a sizable piece of nodular calcium that we successfully fractured using a scoring balloon, thus enabling a successful left main coronary angioplasty. Cutting balloons, scoring balloons, rotational and orbital atherectomy, intravascular lithotripsy (IVL), and excimer laser are the known modalities to treat coronary calcium. Given the limited evidence supporting the use of IVL for breaking nodular calcium [2], we attempted to fracture the calcium nodule in the LMCA using a scoring balloon. This approach resulted in successful calcium elimination, facilitating coronary angioplasty and yielding positive angiographic and clinical outcomes, including distal thrombolysis in myocardial infarction grade III (TIMI III) flow.

## Case presentation

**Figure 1:**
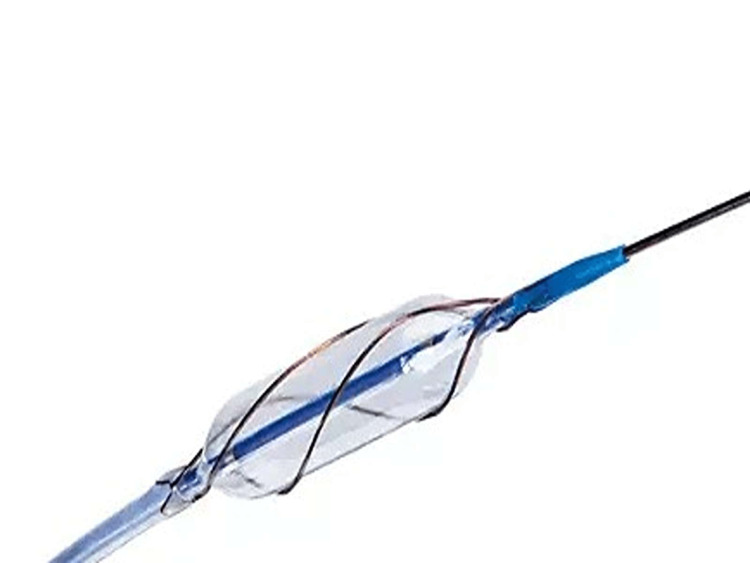
Scoring balloon with three spiraling nitinol struts. Image credit: Dibyasundar Mahanta.

**Figure 2:**
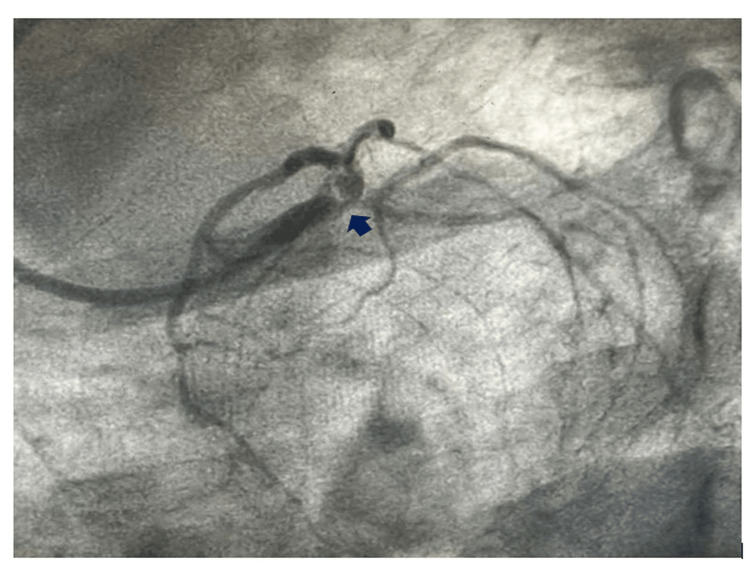
Rectangular nodular calcium in the LMCA bifurcation causing critical coronary occlusion. LMCA, left main coronary artery

**Figure 3:**
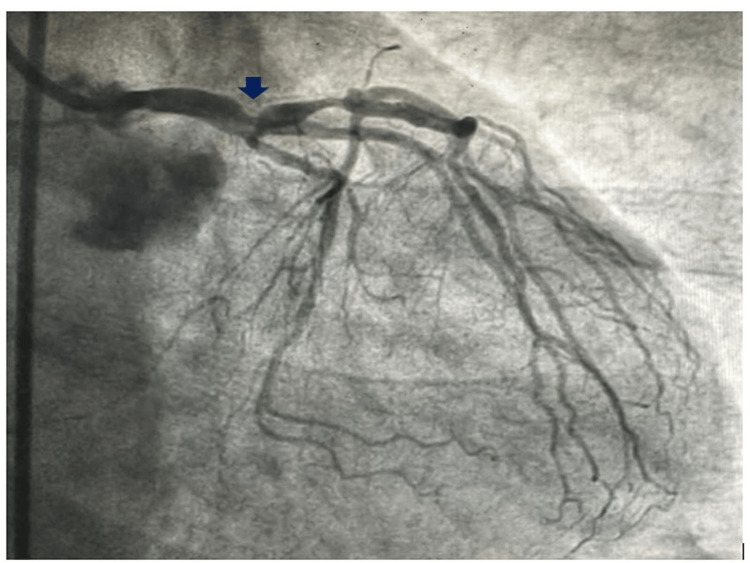
AP view showing nodular calcium in the LMCA bifurcation. LMCA, left main coronary artery; AP, anteroposterior

**Figure 4:**
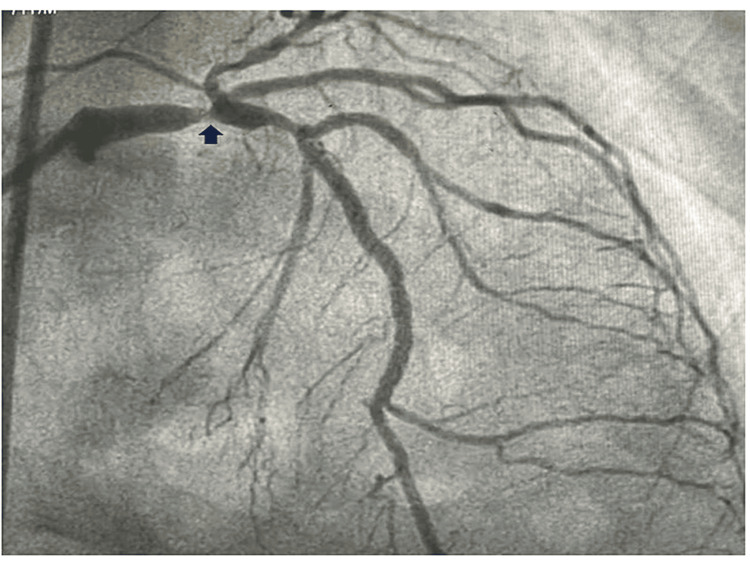
AP cranial view showing nodular calcium in LMCA bifurcation. LMCA, left main coronary artery; AP, anteroposterior

**Figure 5:**
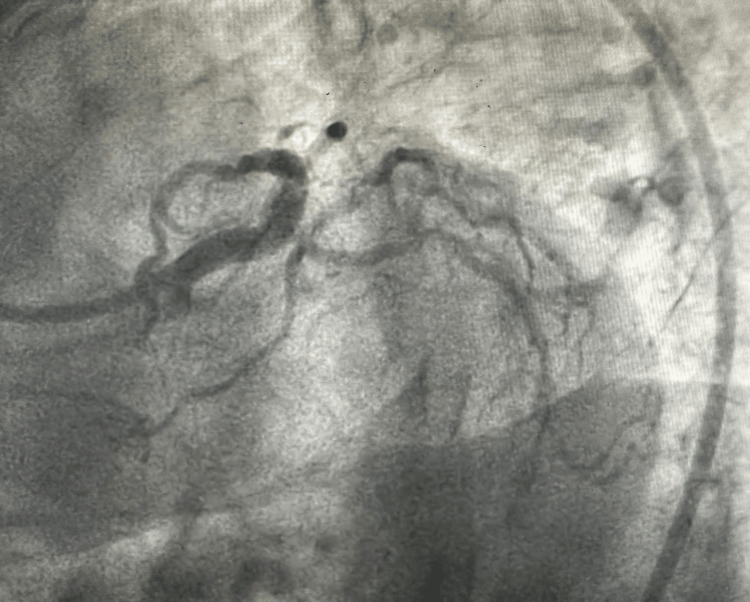
Spider view (LAO caudal) showing good angiographic results post-procedure. LAO, left anterior oblique

**Figure 6:**
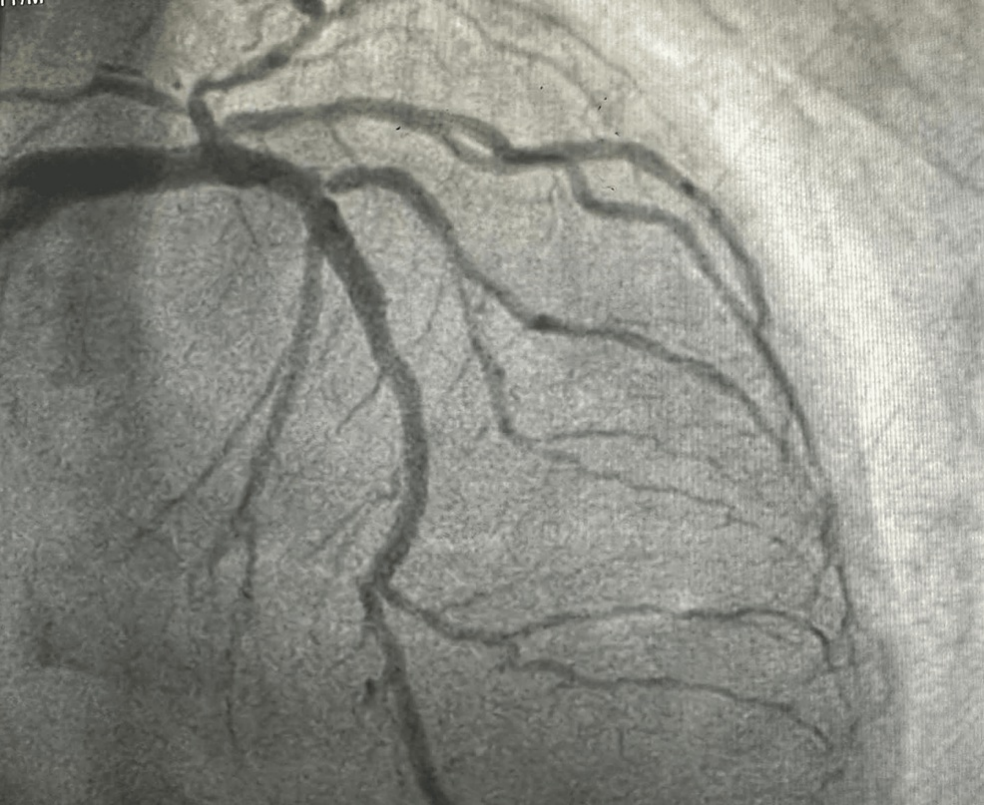
AP caudal view showing good post-procedural angiographic results in LMCA. LMCA, left main coronary artery; AP, anteroposterior

An 80-year-old female presented to the cardiology emergency with angina for one hour, profuse cold diaphoresis, and shortness of breath. She was hemodynamically unstable with a blood pressure of 80/40 mmHg in the right arm supine position with a pulse rate of 130 beats per minute. She exhibited tachypnea and bibasal crepitations, with SpO2 of 88% on room air. The EKG displayed left main coronary artery (LMCA) occlusion, characterized by ST elevation in aVR and reciprocal sagging ST depression in all other leads. She was put on noninvasive ventilation (NIV) and inotrope support. A loading dose of ticagrelor (180 mg) was administered, and the coronaries were injected into the catheterization lab for primary percutaneous intervention (PCI). Coronary angiography revealed occlusion of the LMCA with nodular calcium (Figures 1-4) at the bifurcation with 90% luminal occlusion. The lesion in the LMCA was crossed with a 0.014-inch Fielder FC wire, and the nodular calcium was cracked with a 2.5 mm × 10 mm scoring balloon at 14 atm pressure followed by dilatation with noncompliant (NC) balloons of 3 mm × 10 mm and 3.5 mm × 10 mm at 18 atm, respectively. After balloon dilatation with a scoring balloon, the nodular coronary calcium cracked, yielded, and resulted in the expansion of the minimal luminal area (MLA). The patient experienced hemodynamic improvement, with a reduction in inotropic requirements and an improvement in oxygen saturation. After balloon angioplasty, the lesion was stented from the mid-LMCA to the mid-left anterior descending (LAD) coronary artery with a 4 mm × 34 mm drug-eluting stent (DES) followed by dilatation with a 4.5 mm × 6 mm NC balloon. After percutaneous transluminal coronary angioplasty (PTCA), there was a good angiographic result with distal TIMI III flow (Figures 5-6). The patient was hemodynamically stable off the inotropes with normal room air oxygen saturation. The patient was advised post-procedure anticoagulation with weight and age-adjusted heparin for one day and was discharged in stable condition. Although many centers break the coronary calcium initially with an NC balloon, the index case was attempted to yield the nodular calcium with a scoring balloon followed by NC balloons, which facilitated the left main coronary angioplasty with nodular calcium. Due to the patient's hemodynamic instability despite multiple inotropes, acute left ventricular failure (LVF), bilateral coarse crepitations, and desaturation, coronary imaging was not pursued during or after the procedure, which would have otherwise delayed the procedure and resulted in an unfavorable post-procedure outcome.

## Discussion

Coronary calcium and thrombus are the two main obstacles to successful coronary interventions. During the process of development and maturation of atherosclerotic plaque in coronary arteries, calcium gets deposited inside the atherosclerotic plaque, making it hard, and those calcium spurs protrude into the coronary lumen like big mountains into the lumen of the coronary artery. When they reach a certain size, those calcium spurs are referred to as calcium nodules. A large chunk of calcium in the form of calcium nodules creates difficulty in the passage of coronary hardwires, including balloons and wires, and carries the risk of local vessel dissection and rupture and inadequate stent expansion with future risk of thrombosis and restenosis. Different modalities of debulking the coronary calcium involve the use of an NC balloon, scoring balloon, cutting balloon, rotational atherectomy, orbital atherectomy, IVL, and excimer laser therapy. The scoring balloon consists of a semi-compliant balloon surrounded by an external helical scoring edge. The scoring edge consists of three rectangular spiral indents (Figure 1) and breaks the calcium nodule facilitating coronary angioplasty. It is easy to use, especially during the intervention of critically stenosed patients with hemodynamic instability. The use of more gadgets, including rotaablation, orbital atherectomy, and excimer laser, in hemodynamically unstable critically ill patients increases the procedure time and jeopardizes the clinical outcome. Initial lesion preparation with a scoring balloon followed by a drug-coated balloon (DCB) carries a promising role during the intervention of in-stent restenosis (ISR) [3]. Scoring balloons produce elegant radial cuts, prevent balloon slippage and plaque shift, and achieve adequate expansion of the coronary lumen for successful stent implantation [4]. Scoring balloons minimize the geographic miss [5]. Three spiraling nitinol struts in a scoring balloon offer a mechanical advantage over the noncompliant balloon in breaking the nodular calcium in coronary arteries. The drug-coated scoring balloon provides an advantage over a DCB in treating fibrocalcific ISR [5]. Debulking a coronary lesion with a scoring balloon reduces the target lesion revascularization (TLR), major adverse cardiac event (MACE), and stent thrombosis due to adequate stent expansion [5]. The Grip scoring balloon is another modality of scoring balloons with multiple-point mechanical compression and is also used to treat ISR [6]. For lesion preparation, a scoring balloon is used with the balloon:artery ratio of 0.8 [7], and the most important advantage of a scoring balloon is that it prevents melon seeding (slippage of a balloon out of the target coronary lesion) while preparing a densely calcified lesion [7]. Our case is an interesting illustration of cracking the nodular calcium with a scoring balloon, which is the simplest and safest maneuver to break the nodular calcium during the left main coronary intervention with hemodynamic instability. Scoring balloons equipped with a firm nitinol spiral wire wrap can effectively fracture extensive deposits of nodular calcium in calcified coronary arteries, thereby facilitating coronary angioplasty. Although many modalities to treat coronary calcium are adopted by enthusiastic interventional cardiologists, scoring balloons play a pivotal role in breaking the nodular calcium while addressing critical coronary lesions with hemodynamic instability.

## Conclusions

LMCA nodular calcium poses a great challenge during coronary intervention, especially when the patient is experiencing hemodynamic instability, desaturation, or episodes of ventricular tachycardia. Although rotaablation is the preferred modality of treatment of nodular calcium, in patients with acute myocardial infarction with hemodynamic instability, it plays a limited role. The role of IVL during PCI in acute myocardial infarction is also not established. A simple scoring balloon can crack a large chunk of nodular calcium. This approach is safer, simpler, and faster, facilitating the restoration of antegrade flow even in cases of hemodynamically unstable lesions in the elderly, particularly in the left main coronary artery.
